# The impact of the emoTICare program on socioemotional adjustment and psychological well-being in adolescents with type 1 diabetes mellitus

**DOI:** 10.3389/fendo.2025.1668398

**Published:** 2025-10-03

**Authors:** Javier Martín-Ávila, Esther Rodríguez-Jiménez, Selene Valero-Moreno, José Antonio Gil-Gómez, Inmaculada Montoya-Castilla, Marián Pérez-Marín

**Affiliations:** ^1^ Department of Personality, Evaluation and Psychological Treatments, Faculty of Psychology, University of Valencia, Valencia, Spain; ^2^ Department of Computer Systems and Computation, Higher Technical School of Industrial Engineering. Polytechnic University of Valencia, Valencia, Spain

**Keywords:** quality of life, diabetes mellitus, adolescence, serious game, psychological adjustment, psychological intervention, technological platform

## Abstract

**Introduction:**

Type 1 diabetes mellitus (T1DM) is a chronic disease that can affect the emotional well-being and quality of life of adolescents. This group faces psychosocial and emotional challenges in addition to disease management, making it essential to improve psychological adjustment, emotional regulation, and social skills. The study aimed to explore psychosocial and emotional characteristics of adolescents with T1DM to justify psychological interventions, and to implement a serious game called *emoTICare* to promote clinical and socioemotional health indicators through a technological platform with artificial intelligence. The hypothesis proposed that adolescents would improve clinical and socioemotional indicators after following the *emoTICare* program.

**Method:**

The design was quasi-experimental, single group, pre-post. Seventy-three participants were enrolled, and the final pilot sample comprised 44 Panamanian adolescents with T1DM, 64.4% female. Assessments occurred at baseline (T1), after 6 weeks without intervention (T2), and after the 6-week *emoTICare* intervention (T3), measuring health-related quality of life (HRQoL), disease threat perception, psychopathology, social skills, resilience, self-concept, and emotional awareness. Analyses included repeated measures (ANOVA and Friedman’s Test), comparative tests (Student’s t-test, Mann–Whitney U), correlational analyses, and descriptive statistics.

**Results:**

The initial assessment showed adolescents with high perception of disease threat and reduced quality of life. Boys reported greater physical (p <.01, d=.859), psychological and academic well-being (p <.05), and more adaptive problem-solving (p <.01). After the *emoTICare* intervention, we observed a significant reduction in perceived illness threat (p <.01, ηp²=.145) and improvement in verbal exchange of emotions (p <.01, W=.117). There was also a tendency toward improvements in resilience, self-concept, social skills, and adaptive coping scores.

**Discussion:**

Findings highlight psychosocial vulnerabilities of adolescents with T1DM and show the positive effects of *emoTICare*, particularly in reducing perceived disease threat. The program demonstrates potential as a useful tool for promoting health education and psycho-emotional skills.

**Clinical trial registration:**

ClinicalTrials.gov, identifier EmoTICare NCT06331429.

## 1 Introduction

Diabetes is one of the most prevalent chronic diseases (CD) worldwide (1). Type 1 diabetes mellitus (T1DM) typically manifests during childhood and adolescence, although the onset can occur at any age (2–5). It has been identified as the most prevalent endocrine disorder in children and one of the most common during adolescence (6, 7). The condition is an autoimmune disease marked by the destruction of β cells in the pancreas, which results in absolute insulin deficiency (8). In 2021, the estimated number of cases of T1DM in children under 15 years of age was 651,700, with 108,200 new diagnoses occurring annually. This reflects a growing trend that is driven by high incidence and lower mortality (9).

In the absence of a definitive cure, the treatment of T1DM is based on rigorous blood glucose control, insulin administration, diabetes education, healthy eating, physical exercise, and, in some cases, complementary pharmacological treatment (10, 11). Adherence to treatment is paramount in preventing long-term complications, which have the potential to affect multiple organs and systems (12, 13).

Adolescence is a period characterized by profound transformation, during which a range of physiological, emotional, and social changes occur (14) and where personal identity and autonomy are progressively built (15). The experience of living with a chronic disease, such as T1DM, during this period can present significant challenges, impacting quality of life and leading to difficulties in adhering to treatment regimens (6). In fact, some studies indicate that adolescents have, on average, poorer glycemic control than other age groups (16, 17). Conversely, the presence of psychosocial difficulties has been demonstrated to impede emotional adjustment to the disease, therapeutic adherence, and diabetes management (18, 19), increasing the risk of depressive symptoms, anxiety, and family conflicts (20, 21). It is estimated that more than 25% of children and adolescents with T1DM have comorbid mental disorders (22), which negatively affects their emotional well-being, quality of life, and glycemic control (8, 23).

In this context, psychoeducational interventions targeting adolescents with T1DM have demonstrated efficacy in enhancing health-related quality of life (24, 25), and physical, emotional, and social well-being (26, 27). These approaches tend to prioritize pivotal domains such as psychological adjustment to illness, emotional intelligence and management, enhancement of problem-solving abilities, mitigation of dysfunctional thought patterns, cultivation of an enhanced self-concept and social adeptness, and the curtailment of anxiety, depressive, and behavioral manifestations.

The present study aims to evaluate the effects of an intervention designed for adolescents with T1DM, implemented through the use of a serious game. In recent years, serious games—defined as games or activities focused on achieving a purpose beyond the challenge contained in the game itself, teaching or providing different skills to the player—have gained popularity (28, 29). They have established themselves as a promising tool in the field of health. In the context of our study, it has been observed that games developed specifically for children and adolescents with T1DM promote meaningful learning and are effective in conveying knowledge about the disease, improving self-care skills, and fostering self-efficacy and motivation. These aspects are fundamental to better adjustment to diabetes (30).

Recently, a number of psychoeducational interventions have been published with the objective of enhancing the clinical and psychological well-being of adolescents diagnosed with T1DM. Systematic reviews, such as that by Luque et al. (31) conclude that many of these interventions do not produce significant variations in objective measures of T1DM control, such as glycated hemoglobin (HbA1c). However, these interventions can be useful in improving adolescents’ psychosocial well-being and perception of their disease.

It is imperative to emphasize the distinguishing features of the innovation, progress and clinical contribution of emoTICare in relation to existing interventions. The design of emoTICare is founded on an analysis conducted by our research group of the primary interventions that have emerged during the last 15 years with a focus on enhancing the physical and psycho-emotional well-being of adolescents diagnosed with type 1 diabetes mellitus (32). These interventions were examined in depth, with particular emphasis on analyzing their design, the underlying theoretical model and the inclusion of five important indicators/variables of health-related quality of life among the areas of work of these interventions (physical well-being, cognitive coping, emotional well-being, social relationships and support, and identity). A review of the extant literature revealed that the majority of these interventions were devoid of a robust and generalizable theoretical foundation, as they approached the issue from a plethora of perspectives, with a paucity of clarity regarding the specific domains in which these interventions were designed to promote enhanced health-related quality of life and effective adaptation to the disease. The observations revealed that 50% of the cases included only three of the areas, 23.6% addressed four of them, 18.42% addressed two areas, and 7.8% addressed only one of them. Conversely, we can emphasize how emoTICare is predicated on a robust theoretical model, the Disease Adjustment Model from an Integrative Perspective (DAMIP) (33), which incorporates the interaction of the main relevant factors that determine adequate adaptation to chronic disease in adolescence. Furthermore, the six missions of emoTICare embody a holistic strategy encompassing psychoeducation and clinical adaptation to diabetes, in addition to the enhancement of psychosocial and emotional competencies. This multifaceted approach integrates all five areas mentioned above into its intervention components, an innovative aspect that allows for a greater and much more comprehensive scope in adjusting to the illness and improving the psycho-emotional health of these adolescents. The emoTICare system is presented through an intelligent technological platform that incorporates artificial intelligence, representing a highly innovative application in this field.

(32, 33)The primary objective of the study was to explore the psychosocial and emotional characteristics of a sample of adolescents with T1DM. The secondary objective was to justify the importance of psychological interventions in this population, to then implement a serious game called emoTICare, that aims to promote clinical and socioemotional health indicators, as well as the development of socio-emotional skills among adolescents with T1DM through a psychological intervention conducted on a technological platform that incorporates artificial intelligence. The primary outcomes were health-related quality of life (HRQoL), emotional and behavioral problems, coping/problem-solving strategies, emotional competences, self-concept, and social skills. Secondary outcomes included perceived illness threat, and resilience. The study hypothesizes that adolescents will show improvements of these clinical and socioemotional health indicators after following the emoTICare program.

## 2 Method

### 2.1 Description of the sample

The sampling method that was selected was convenience sampling. In order to be considered for inclusion in this study, participants were required to meet the following criteria: first, they had to be between 12 and 16 years of age; second, they had to have been diagnosed with type 1 diabetes mellitus (T1DM) at least six months prior to the diagnosis; and third, they had to attend the pediatric endocrinology outpatient service at a hospital on a regular basis for medical follow-up.

The following exclusion criteria were established: a) diagnosis of cerebral palsy or epilepsy; b) presence of brain tumors; c) psychological diagnosis prior to the onset of the organic disease.

**Figure 1:**
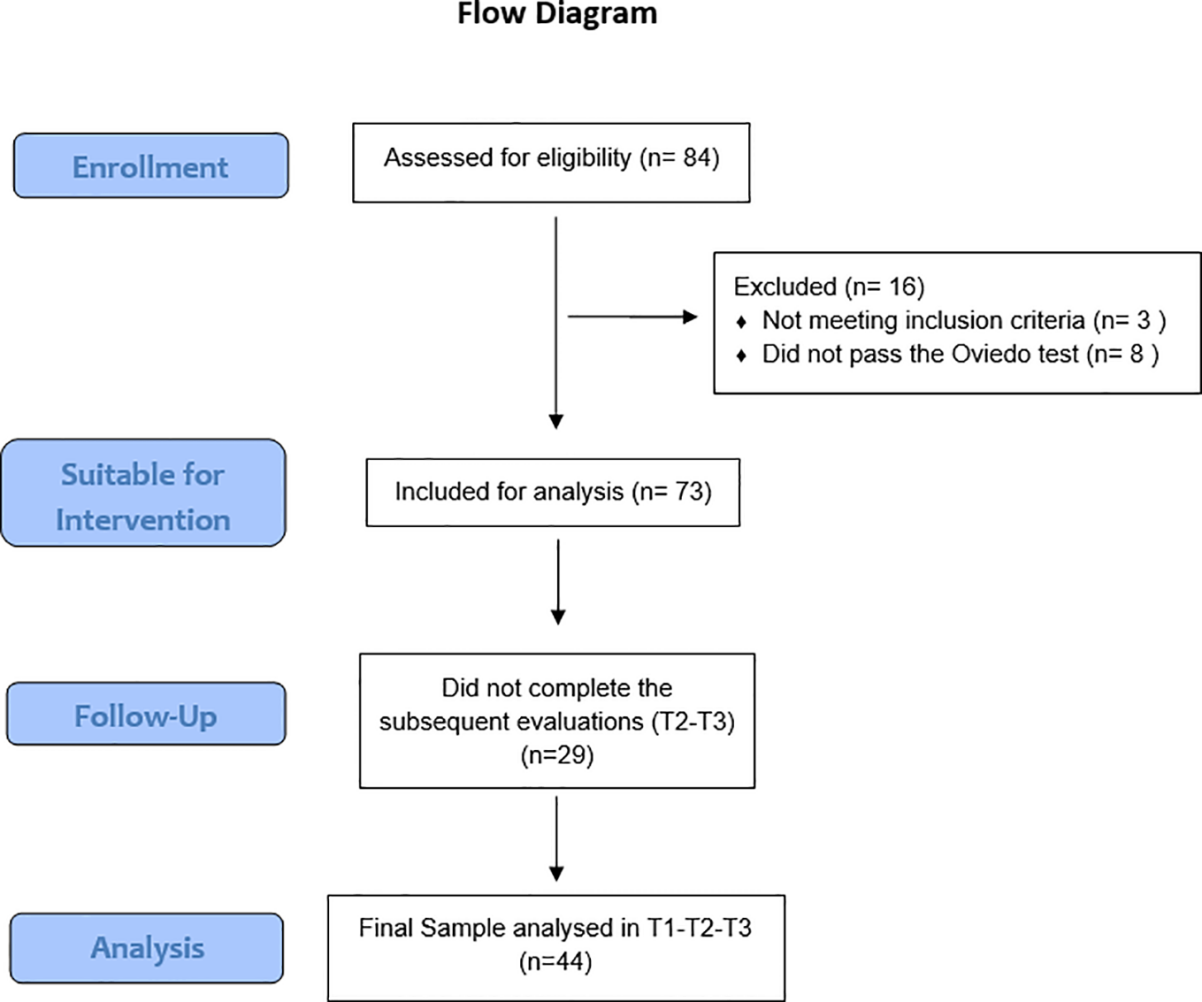
CONSORT flow diagram.

The study sample was recruited through DiabetesLATAM, a non-governmental organization and foundation headquartered in Panama with outreach across Latin America. The principal mission of this organization is to provide education and support to individuals diagnosed with type 1 diabetes and their families. Participant recruitment was conducted between June and November 2024.The flowchart of the intervention protocol followed in the research is shown below (see **Figure 1**).

### 2.2 Design and procedure

To calculate the sample size, we conducted an *a priori* power analysis using the software G*Power 3.1 (34), for a repeated-measures ANOVA (within-subjects factor: time; 3 levels: T1, T2, T3), with α = .05 and power (1–β) = .95. We specified a medium effect size (ηp² = 0.06) following Cohen’s conventions (35), as adopting a medium effect size balances realism and clinical relevance, avoiding under- or overestimation of the intervention’s potential. We further assumed a correlation among repeated measures of r = .50 (consistent with psychosocial outcomes over short-term follow-ups). Under these assumptions, the required sample size was N = 46. Considering expected attrition in adolescent sample, we initially enrolled a larger number of participants (N = 84). Finally, as stated in the Consort Flow Diagram (**Figure 1**), the final analyzable sample was N = 44.

The intervention was meticulously designed as follows: through the DiabetesLATAM association, information about the emoTICare project was disseminated to its partners and members so that, on a voluntary basis, the legal guardians of interested parents or adolescents could contact the research team to express their interest in participating in the study.

**Figure 2:**
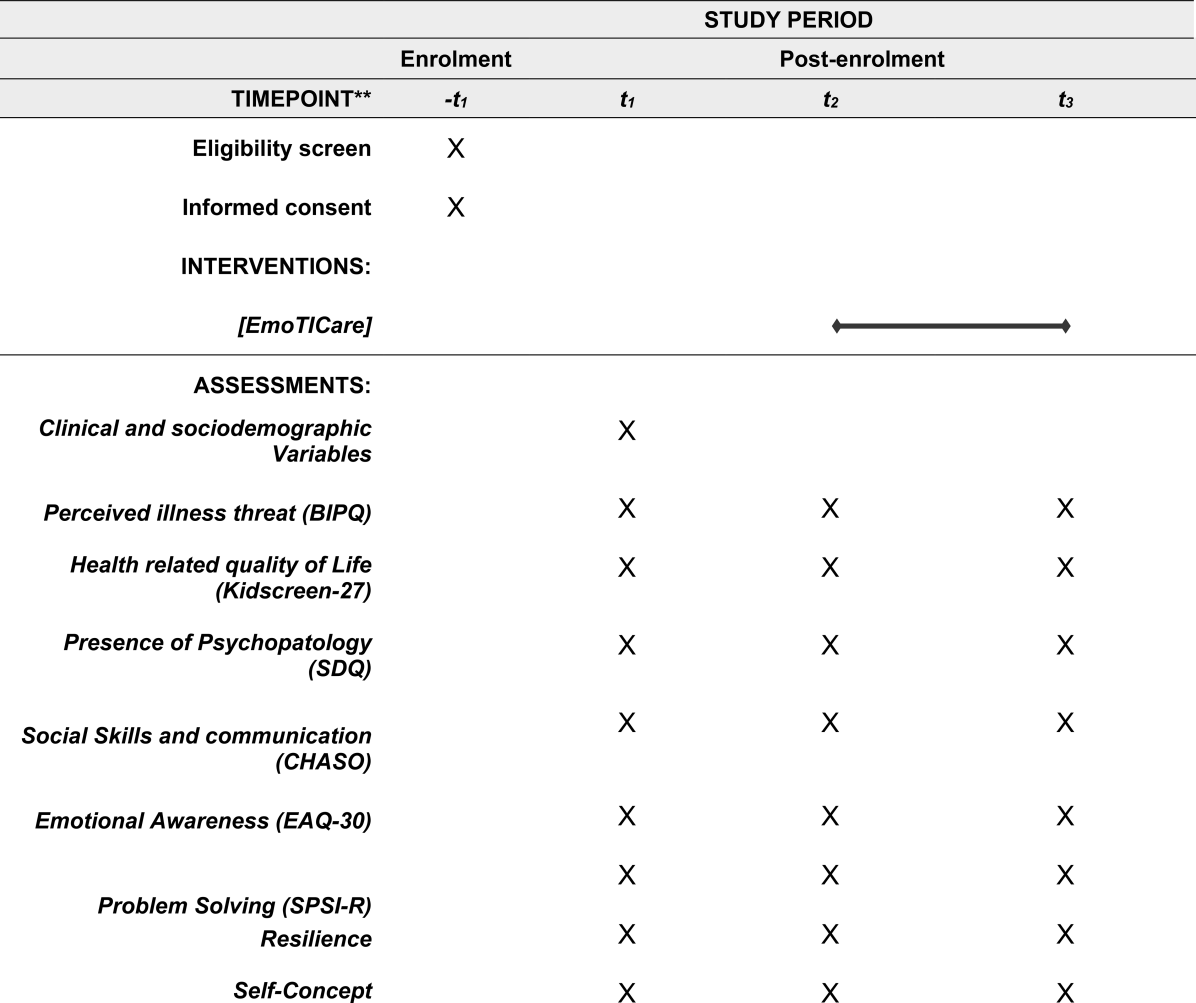
SPIRIT schedule of enrolment, intervention and assessment.

Following the receipt of information regarding the project by the adolescents’ guardians and the provision of their informed consent, as well as that of the minors under their care, a researcher disseminated the link to the assessment to the parents/legal guardians of the participants. The assessment was conducted online via Limesurvey, a complimentary platform designed for the creation and administration of surveys and questionnaires (T1). Therefore, the responsibility falls upon the parents/legal guardians to furnish the minors with the links to the three assessments and the emoTICare application. Initially, immediately after completing the informed consent online, a first evaluation was conducted for all participants (T1), covering the main areas that constitute the psycho-emotional state of the participants. Following this preliminary evaluation, the subjects were reassessed after six weeks and subsequently initiated the intervention, which spanned a duration of six weeks. After the participants completed the emoTICare serious game, a third assessment (T3) was carried out on all participating adolescents. Assessments T1 and T2 functioned as a control measure for all participants in relation to the variables evaluated. The present study exclusively examined alterations that occurred naturally over time without external intervention. In T3, conducted after the intervention, the benefits achieved in these variables as a result of the emoTICare intervention were analyzed. The complete enrollment and evaluation schedule can be found in **Figure 2**.

emoTICare is a serious game that was developed with the specific purpose of serving adolescents who have been diagnosed with type 1 diabetes mellitus (T1DM). The model is grounded in the fundamental principles of the Disease Adjustment Model from an Integrative Perspective (DAMIP) (33), which integrates the main theoretical components of Livneh’s Integrative Model (36, 37), Antonovsky’s Salutogenic Model (38), Leventhal’s self-regulation model of illness (39), and the Health Belief Model (40, 41). This approach enables a multidimensional examination of the adaptation process to the disease, encompassing cognitive, emotional, behavioral, and contextual factors. The DAMIP model identifies five key areas when addressing proper adaptation to chronic disease in adolescence. The aforementioned domains encompass physical well-being, emotional well-being, cognitive coping, identity and social relationships, and support.

emoTICare is characterized by a graphic adventure narrative (for a more detailed exposition of the structure and content of the serious game, consult the protocol article by Martín-Ávila et al. (42), in which the user is tasked with completing six missions that are associated with a variety of scenarios and themes. These missions are designed to reinforce the key areas identified by the DAMIP model. Each mission corresponds to a pivotal domain encompassed by the theoretical framework and the intervention, namely: (a) psychoeducation concerning T1DM; (b) emotional awareness; (c) emotional regulation; (d) cognitive coping; (e) identity and self-concept; and (f) social skills and communication. The activities within the game combine interactive dynamics, psychoeducational techniques, and challenges linked to the everyday context of adolescents with T1DM. This combination fosters meaningful learning and the transfer of skills to their daily lives.

The implementation of the “emoTICare” intervention program in this pilot sample of Panamanian nationals followed a quasi-experimental, single group, pre-post design, that uses descriptive, correlational, and comparative methodology. This study adheres to the ethical guidelines of the 2013 Declaration of Helsinki of the World Medical Association and has been approved by the Human Research Ethics Committee of the University of Valencia (Reference 2023-PSILOG-3178945, UVINV_ETICA-3178945). Appropriate measures have been taken to ensure the complete confidentiality of participants’ data, in accordance with Organic Law 3/2018, of December 5, on the Protection of Personal Data (LOPD). This study and its protocol have been approved and registered as a clinical trial in the ClinicalTrials.gov PRS (Protocol Registration and Results System) (Reference - ID: emoTICare NCT06331429). In addition, copyright: © emoTICare registration number: UV-SW-202481R © University of Valencia & Polytechnic University of Valencia, 2024. All rights reserved.

### 2.3 Analyzed variables

In order to evaluate the different variables related to the performance of emoTICare in adolescents with T1DM, the following battery of computerized questionnaires was developed:

#### 2.3.1 Clinical and sociodemographic variables

Through an *ad hoc* registry, and after obtaining informed consent, the variables of gender, age, and previous contact with psychology professionals were evaluated, as well as the reason for consultation.

Likewise, with the aim of gathering as much relevant data as possible related to the disease and its impact on adolescents, information on the following medical/clinical variables was also collected through an *ad hoc* registry:

- Cause and number of hospitalizations due to diabetes
- Months since the onset of the disease
- Degree of difficulty perceived by the disease
- Specification of what is related to diabetes that causes the greatest difficulty
- Presence of other medical diseases
- Presence of psychological disorders
- Type of medical treatment they are receiving for T1DM
- Clinical consequences of T1DM

Among the main items of interest of this study of this study were to access clinical indicators of health from the target population of adolescents with T1DM. We recorded self-reported clinical indicators/variables related to physical well-being and illness perception, including perceived threat of illness, and certain subjective clinical consequences associated with the condition (e.g., vision difficulties, pain in hands or feet, urination problems, problems with digestion). This approach enabled the assessment of relevant health-related experiences despite the absence of direct physiological indicators.

#### 2.3.2 Psychological variables

The psychological variables mentioned below, as well as the measurement instruments used for their assessment, are described in greater detail in the description of the different areas that make up emoTICare:

- Perception of illness threat ➔ We used the *Brief Illness Perception Questionnaire* (BIP-Q) developed by Broadbent et al. (43), in its shortened version validated in a sample of Spanish-speaking adolescents (Brief Illness Perception Questionnaire, BIP-Q) (44). This is an abbreviated version of the Illness Perception Questionnaire (IPQ) (45), consisting of five items with Likert-type responses (0–10) based on the degree of agreement, plus a final item with an open-ended response option. Higher scores indicate that the subject manifest a higher perception of illness threat. This questionnaire assesses cognition of illness through the factors: “Consequences of the disease,” “Duration of the disease,” and “Identity,” as well as emotion through the subscales “Concern about the disease” and “Emotional impact of the disease”. The scale has been successfully validated in Latin America (46). In our sample, this scale obtained a reliability coefficient of α=.756.
- Health Related Quality of Life ➔ Validated version adapted to Spanish-speaking samples by the Kidscreen group from the KIDSCREEN-27 questionnaire (47). This instrument consists of 27 items with Likert-type responses (1–5), divided into five dimensions: ‘*Physical Well-Being”* (5 items) assesses activity, energy, fitness, and health (low: exhaustion and poor health; high: fitness, energy, and good health); *“Psychological Well-Being”* (7 items) covers emotions, life satisfaction, and absence of sadness (low: unhappiness, depression, low self-esteem; high: happiness, balance, and positive outlook); “*Autonomy and Parent Relations”* (7 items) evaluates family support, autonomy, and financial resources (low: restriction, neglect, financial strain; high: supportive relationships, autonomy, and financial well-being); “*Social Support and Peers”* (4 items) reflects peer relationships (low: exclusion; high: acceptance and support); “*School Environment”* (4 items) assesses learning, concentration, and school relationships (low: dislike of school, negative attitudes; high: enjoyment of school and good adjustment). Some items are negatively worded and must be reverse-scored to ensure consistency in directionality. This questionnaire has shown solid psychometric properties among Spanish-speaking adolescents (47, 48). For our sample, the reliability of the subdimensions was as follows: “physical well-being” α=.789; “psychological well-being” α=.863; “autonomy and relationship with parents” α=.811; “social and peer support” α=.811; and “school environment” α=.745.
- Presence of psychopathology ➔ We used the *Strengths and Difficulties Questionnaire* (SDQ) developed by Goodman (49), in its Spanish version (Strengths and Difficulties Questionnaire, SDQ) validated by Ortuño-Sierra et al. (50). It consists of 25 items with Likert-type responses (0–2), grouped into 5 dimensions or scales, with 5 items each (range 0–10 for each subdimension): “Emotional Symptomatology”, “Behavioral Problems”, “Hyperactivity, Peer Relationship Problems”, and “Prosocial Behavior”. Higher scores reflect greater difficulties in each subdimension, except for “Prosocial Behavior,” where higher scores indicate fewer problems. Some items are negatively worded and must be reverse scored to ensure consistency in directionality. The questionnaire has also been validated in the Latin American population (51). In this sample, Cronbach’s α was α=.505 for “hyperactivity,” α=.814 for “emotional symptoms,” α=.477 for “peer problems,” α=.669 for “prosocial behavior”; α=.558 for “behavioral problems”; and α=.779 for the total difficulty scale.
- Social skills and communication skills ➔ Specifically in its latest version validated in a Spanish-speaking sample by Caballo et al. (52) of the Social Skills Questionnaire (CHASO). This instrument assesses ten subdimensions of social functioning, each representing a distinct aspect of interpersonal behavior, and consists of 40 items rated on a five-point Likert scale, with each subdimension represented by four items. “Interacting with strangers” reflects the ability to initiate and maintain conversations with unfamiliar people, “expressing feelings” evaluates the capacity to communicate emotions openly and appropriately, “coping with criticism” refers to responding constructively and assertively when receiving negative feedback, “interacting with people one is attracted” to assesses confidence and adequacy in approaching and conversing with potential partners, “remaining calm in the face of criticism” captures emotional self-control and regulation when confronted with disapproval, “speaking in public” measures the ability to communicate effectively before an audience and manage performance anxiety, “coping with ridicule” examines resilience and adaptive responses when facing mockery or teasing, “defending one’s rights” reflects assertiveness in protecting personal boundaries and interests in social or professional contexts, “apologizing” evaluates the willingness and skill to acknowledge mistakes and repair interpersonal relationships, and “rejecting requests” refers to the ability to say no without guilt or excessive anxiety while maintaining social appropriateness. Higher scores reflect greater ability in the respective subdimension. The overall reliability of the scale was α=.920. For the subdimensions, Cronbach’s α was: α=.768 for “interacting with strangers,” α=.898 for “expressing feelings”; α=.860 for “coping with criticism,” α=.872 for “interacting with people you are attracted to”; α=.753 for “remaining calm in the face of criticism”; α=.661 for “speaking in public”; α=.568 for “coping with ridicule”; α=.644 for “defending rights”; α=.874 for ‘apologizing’; and α=.874 for “rejecting requests.”
- Emotional awareness ➔ We used the *Emotion Awareness Questionnaire* (EAQ-30) developed by Rieffe et al. (53), in its validated version adapted into Spanish by Samper-García et al. (54). It consists of 30 items rated on a three-point Likert scale (“not true,” “sometimes true,” “often true”), organized into six subdimensions of emotional awareness: “distinction of emotions”, which assesses the ability to distinguish between different emotional states, “verbal exchange of emotions”, which evaluates the capacity to communicate feelings to others, “non-concealment of emotions”, which reflects openness and authenticity in expressing emotions, “body awareness”, which measures sensitivity to the physical sensations associated with emotions, “emotions of others”, which captures empathy and the recognition of emotional cues in others, and “analysis of emotions”, which evaluates the ability to reflect on and understand the causes and consequences of one’s emotional experiences, together providing a multidimensional framework for assessing the development of emotional awareness in youth populations. Some items are negatively worded and must be reverse scored to ensure consistency in directionality. The validation of the general scale in a Spanish sample obtained a score of α=.74. This scale has also been used and validated in Latin America, where it obtained reliability and validity values similar to those obtained in the Spanish sample (55). For our sample, the reliability of the subdimensions was as follows: α=.775 for “distinction of emotions”; α=.710 for “verbal exchange of emotions”; α=.645 for “non-concealment of emotions”; α=.070 for “body awareness”; α=.708 for “analysis of emotions”; α=.512 for “emotions of others” and α=.720 for the total scale.
- Coping skills and problem solving ➔ short version of the Social Problem-Solving Inventory – Revised (SPSI-R) questionnaire by Zurilla and Nezu (56). This instrument attempts to reflect cognitive, affective, and behavioral responses to everyday problems or various difficulties. It consists of 25 items with Likert-type responses (0-Not true in my case, 4-Totally true in my case), grouped into 5 dimensions composed of 5 items each: “positive problem orientation” (constructive attitude and confidence in problem solving), “negative problem orientation” (viewing problems as threats with self-doubt and frustration), “rational problem-solving” (systematic and effective strategy use), “impulsivity/carelessness style” (hasty and inattentive approaches leading to errors), and “avoidance style” (procrastination, denial, or shifting responsibility). A high score for the various subdimensions highlights the extent to which these styles influence their problem solving abilities. This instrument has been validated in a Latin American sample by Merino (57).. The reliability for the subdimensions of this scale was: α=.781 for “rational problem solving”; α=.632 for “avoidant problem-solving style”; α=.727 for “impulsive problem-solving style”; α=.786 for “positive orientation toward problem solving”; and α=.822 for “negative orientation toward problem solving.”
- Resilience ➔ We used the 10 items reduced version of *Connor-Davidson Resilience Scale* (CD-RISC) (58) in its Spanish version, validated by Notario-Pacheco et al. (59). The test yields a unidimensional structure that captures core aspects of resilience such as adaptability, sense of purpose, self-efficacy, emotional regulation, and the capacity to recover from difficulties, providing a reliable and efficient global indicator of psychological resilience. This instrument consists of 10 items with Likert-type response options (0-Not at all, 4-Always) and whose psychometric properties have been shown to be correct (α=.85). This scale has also been validated in Latin America, where the unifactorial structure of the original questionnaire has been confirmed and adequate psychometric properties have been obtained (α=.82) (60). For our sample, the reliability of this scale was α=.863.
- Self-Concept ➔ We used the latest version of the Garley Self-Concept Questionnaire (GSC), elaborated in by García Torres (61). It has 48 Likert-type response items (1-Never, 5-Always), which provide an overall self-concept score, divided into 6 subdimensions composed of 8 items each. This subdimensions are: “physical self-esteem”, that refers to their perceived physical appearance; “social self-esteem”, that tries to measure the degree of social acceptance from their reference group and socialization skills; “family self-esteem”, that measures the degree of perceived acceptance from their family members; “intellectual self-esteem” that refers to their perceived cognitive abilities, specially their school performance; “personal self-esteem” which encompasses different evaluations about themselves and “control capacity”, that attempts to measure their perception of the degree of control they have when acting to obtain certain predictable results. This measurement tool has also shown acceptable psychometric properties in Latin American population with two different samples, where a reliability of α=.85 and α=.90 respectively was obtained (62, 63). In this sample, the subdimensions of the scale obtained a reliability of: α=.778 for “physical self-esteem”; α=.808 for “social self-esteem”; α=.761 for “family self-esteem”; α=.780 for “intellectual self-esteem”; α=.627 for “personal self-esteem, α=.648 for “control capacity” and α=.909 for the sum of all dimensions.

Finally, as a control variable, the Oviedo Response Infrequency Scale protocol, developed by Fonseca-Pedrero et al. (64), comprised of 12 Likert-type items (1–5). With the incorporation of this control variable, we tried to ensure the reliability of the responses given by the different subjects, so that if any of them answered incorrectly to 3 of the items, the responses to the questionnaire would be invalidated.

### 2.4 Statistical analysis performed

Statistical analyses were conducted using the SPSS program, version 28.0 for Windows. The statistical procedures employed to address the hypotheses were as follows:

#### 2.4.1 Descriptive statistics

The descriptive statistics employed included frequency tables (Fr), percentages (%), means (M), percentiles (P), minimums and maximums, and standard deviations (SD).

#### 2.4.2 Spearman’s correlation coefficient

Given that some of the variables did not meet the assumption of normality, Spearman’s correlation coefficient was employed to study the possible correlations between variables.

#### 2.4.3 Saphiro Wilks and Kolmogorov-Smirnov test

To ascertain the normality of the quantitative variables in our study, we employed both the Kolmogorov-Smirnov test, which is the test indicated when dealing with a sample number greater than 50, and the Saphiro Wilks test for samples containing fewer than 50 subjects.

#### 2.4.4 T Student test for independent samples

The study of comparison of means between two independent groups in those variables that met the assumption of normality was performed using the T Student test for independent samples. The homogeneity of variances was analyzed using Levene’s test.

#### 2.4.5 Mann-Whitney U

For comparisons between two independent groups of variables that did not meet the normality assumption, the Mann-Whitney U, a nonparametric test equivalent to the T test for independent samples, was applied. The variables that did not meet the requirements for the Student’s T test are marked in the results section in cursive and their average rank:/*/Ar//.*

#### 2.4.6 Effect size Cohen’s d

Measurement of effect size based on mean differences to measure the relative strength of significance was performed using Cohen’s d, where Student’s t-test was used. Effect sizes were considered low at values below 0.2 and high at values equal to or above 0.8 (Cohen, 1998).

#### 2.4.7 Effect size r

The effect size of the differences observed in the Mann-Whitney U test was reported by the Rosenthal r coefficient, calculated by the following expression: $r=\frac{Z}{\sqrt{N}}$ .

#### 2.4.8 Repeated-measures ANOVA

In order to compare the differences in the scores on the psychological variables under study between the three-time measures (T1, T2, T3), a repeated measures ANOVA was applied to the variables that met the assumption of normality and homoscedasticity.

#### 2.4.9 Friedman test

For those variables that did not meet the assumption of normality and homoscedasticity, the Friedman’s test was used to compare the differences in the scores on the psychological variables under study between the three time points (T1, T2, T3). The variables that did not meet the requirements for the ANOVA test are marked in the results section in cursive and their average rank:/*/Ar//.*

#### 2.4.10 Effect size ηp²

To quantify the practical magnitude of the changes observed across measurements in the repeated measures ANOVA, the partial eta squared (η_p_^2^) was calculated for each variable, indicating the proportion of variance explained by the time effect.

#### 2.4.11 Kendall’s W effect size

It was used to compare the effect size of the observed differences using the Friedman test.

## 3 Results

### 3.1 Intrasubject Analysis of emoTICare benefits

As illustrated in **Table 1**, a comparison of means among the varying measurement times (T1-T2-T3) was conducted to analyze the alterations in the aforementioned psychological variables. This analysis was conducted prior to and following the subjects’ receipt of benefits associated with the utilization of emoTICare. Following the loss of 29 subjects due to dropouts (a common occurrence in longitudinal studies, as previously outlined in preceding sections), the final sample size for this longitudinal analysis was 44.

**Table 1:** Repeated measures ANOVA at the 3 time points.

Questionnaire	Variable	Group			*F/X^2^*	*P (ηp²/W)*	*T1-T2*	*T2-T3*	*T1-T3*
		T1 *//Ar//M (DT)*	T2 *//Ar//M (DT)*	T3 *//Ar//M (DT)*					
BIPQ	Perceived threat of illness	31,70 (9,64)	30,25 (9,50)	27,45 (9,30)	7,321	,001** (,145**)	,687	,018*	,003**
Kidscreen-27	*Physical Well-Being*	*//2//43,34 (11,48)*	*//2,11//44,67 (9,93)*	*//1,89//42,80 (10,06)*	*1,198*	*,549(,014)*	*1*	*,859*	*, 1*
	Psychological Well-Being	42,58 (11,68)	42,76 (12,54)	41,90 (11,84)	,151	,860 (,027)	1	1	1
	*Family Support*	*//1,88//44,34 (9,87)*	*//2//44,15 (10,59)*	*//2,13/44,88 (7,72)*	*1,541*	*,463(,018)*	*1*	*1*	*,723*
	*Social Support*	*//2,16//47,30 (12,03)*	*//1,94//44,85 (11,24)*	*//1,90//45,33 (9,19)*	*2,142*	*,343(,024)*	*,282*	*,934*	*,661*
	*School welfare*	*//2,15/47,56 (9,76)*	*//1,90//46,46 (10,39)*	*//1,95//46,55 (7,63)*	*1,834*	*,400 (,021)*	*,723*	*1*	*1*
CD-RISC	Resilience	22,47(9,47)	22,59 (7,09)	23,25 (6,22)	,185	832 (,004)	1	1	1
SDQ	*SDSE*	*//1,99//4,02 (3,02)*	*//2,17/4,43 (2,70)*	*//1,84//3,70 (2,66)*	*2,890*	*,236 (,033)*	*1*	*,367*	*1*
	*SDQH*	*//2,08//4,59 (1,86)*	*//2,14//4,73 (2,09)*	*//1,78//4,09 (2,06)*	*4,074*	*,130 (,046)*	*1*	*,295*	*,497*
	Total score	14,61 (5,99)	14,75 (6,51)	14,27 (6,28)	,217	,806 (,005)	1	1	1
CHASO	Interacting with strangers	9,66 (3,69)	9,26 (3,75)	9,70 (3,81)	,594	,554 (,011)	1	1	1
	Public speaking	11,52 (3,53)	11,20 (4,08)	11,82 (3,74)	,688	,505 (,016)	1	,511	1
	*Facing ridicule*	*//1,91//9,68 (3,42)*	*//1,95//9,00 (3,45)*	*//2,14//9,66 (3,28)*	*1,493*	*,474 (,017)*	*1*	*1*	*,859*
CAG	Physical self-concept	28.45 (5.07)	28.84 (5.30)	28.65 (6.24)	,150	,861 (,003)	1	1	1
	*Social self-concept*	*//1,89//26.29 (7.13)*	*//2,19//27.22 (6.84)*	*//1,92//26.77 (6.32)*	*2,755*	*,252 (,031)*	*,450*	*,602*	*1*
	*Family self-concept*	*//1,88//28.61 (5.18)*	*//2,22//29.29 (5.79)*	*//1,91//29.50 (4.52)*	*3,250*	*,197 (,031)*	*1*	*,450*	*,329*
	*Intellectual Self-Concept*	*//1,91//26.56 (5.65)*	*//1,98//26.47 (5.78)*	*//2,11//27.52 (5.12)*	*1,057*	*,590 (,012)*	*,337*	*,522*	*,749*
	Control capacity	25.50 (5.35)	24.54 (4.96)	25.77 (4.60)	1,612	,205 (,036)	,601	,301	1
	Total self-concept	162.70 (25.13)	163.93 (25.30)	165.45 (24.16)	,413	,663 (,01)	1	1	,723
EAQ	Distinction of emotions	13.31 (3.29)	13.86 (3.13)	14.43 (3.32)	2,666	,075 (,058)	,791	,743	,076
	*Non-concealment of emotions*	*//1,92//9.15 (2.11)*	*//1,95//9.36 (2.35)*	*//2,13//9.63 (2.46)*	*1,248*	*,536 (,014)*	*1*	*1*	*1*
	*Verbal exchange of emotions*	*//1,66//5.27 (1.82)*	*//2,09//5.79 (1.70)*	*//2,25//6.11 (1.91)*	*10,270*	,006** (,117)	*,128*	*1*	,017*
	Total emotional awareness	62 (6.70)	62.54 (6.63)	64.04 (7.74)	2,355	,101 (,052)	1	,422	,199
SPSI	Positive problem orientation	10.25 (5.12)	9.61 (3.83)	11.22 (3.81)	2,430	,094 (,053)	1	,058	,660
	Negative orientation to the problem	10,05 (5,54)	9.45 (5,20)	9,57 (4,83)	,387	,680 (,009)	1	1	1
	Adaptive coping factor	20.72 (8.48)	19.38(7.39)	21.56 (7.50)	1,555	,217 (,035)	,879	,227	1

//Ar//, Mean Ranges; M, Mean; SD, Standard Deviation; F: ANOVA test statistic; ηp², Partial Eta squared,(0.01=) (weak) (effect) (,0.06*=) (medium) (effect) (,>0.12**=) (large) (effect) ().T1, Time 1; T2, Time 2; T3, Time 3; p, level of significance p ≤0.05* and p ≤0.01**) (); (Kendall’s W, (effect size) (0.1-0.3=weak effect,(0.3-0.5*, medium effect) (,>0.5**=) (large effect).

Prior to conducting the analysis, it was imperative to ascertain that all variables satisfied the assumption of normality. However, upon examination, it was discovered that certain variables did not meet this criterion. Consequently, a decision was made to undertake a mixed analysis contingent upon the fulfillment of this criterion. Consequently, the table presents both the “F” statistic of the ANOVA test and the X2 statistic of Friedman’s nonparametric test. In both cases, the Bonferroni correction method has been employed for the *post-hoc* pairwise test, the objective of which is to ascertain the presence of significant changes between specific time points. The partial eta squared statistic (ηp2) was employed to calculate the effect size in the context of repeated measures ANOVA, while Kendall’s W was utilized to calculate the effect size of the Friedman test. As in the previous contrast, the non-normal variables are identified by the presence of the data of the average ranges, identified with the symbol/*/Ar//.*

Among the results found, we highlight for its significance the statistically significant decrease in the perception of threat of disease between the three-time measurements. A subsequent examination of the *post-hoc* checks reveals that the observed change is statistically significant in both the time intervals between T1 and T3, as well as between T2 and T3. However, no statistically significant change was detected in the interval between T1 and T2, prior to the adolescents’ engagement with emoTICare. The effect size observed in this case can be defined as moderate/high. A positive trend in the results is evident when examining the changes presented in the other variables analyzed, suggesting that the benefits received after the application of emoTICare are significant.

A notable increase in scores was observed in factors such as resilience, which improved between T1 and T2, and between T2 and T3, with a bigger variation in this last time period. Concurrently, a decline in emotional symptomatology and hyperactivity (measured by SDQ) between T2 and T3 was documented, exhibiting moderate and large effect sizes respectively. This decline occurred concurrently with the gaming experience, indicating a positive effect of the gaming experience on the emotional state of adolescents.

With respect to self-concept, the findings suggest a positive trend across all subdimensions of the scale. The T3 scores of all subdimensions of the Garley self-concept scale, as well as the overall self-concept score, have been demonstrated to exceed the initial values. Some of these changes are bigger between T1 and T2 (physical self-concept, social self-concept) while others are bigger in the time period between T2 and T3 (Total Self-Concept, Control Capacity, Intellectual Self-Concept).

A similar upward trend was observed in adolescents’ global emotional awareness (measured by EAQ) following the emoTICare play period. This same positive increase occurred to a greater degree between T2 and T3 in the subdimensions of emotion distinction and non-concealment of emotions and in the total emotional awareness score. Furthermore, it is imperative to acknowledge the significant increase between T1 and T3 in the subdimension of “verbal exchange of emotions,” which was accompanied by a small effect size.

In the context of problem-solving, an enhancement in the positive orientation toward problems between T2 and T3, as well as the adaptive factor toward problems (i.e., the aggregate of positive orientation and rational coping with the occurrence of a problem) also between T2 and T3. Finally, it should be noted that there was a decrease in the degree to which adolescents exhibited a negative orientation in coping with their problems between T1 and T3.

Regarding the adolescents’ personal evaluation of their emotional state (0-I feel very bad; 10-I feel very good) during the emoTICare experience, it is evident that their emotional state has shown significant improvement over time, particularly following the experience itself (**Table 2**). Consequently, the mean of the emotional state increased from 6.89 at T1 to 7.16 at T3, and the difficulties (i.e., difficulties present in their daily life; 0-I have no difficulties or problems; 10-I have many difficulties, my situation is very complicated) decreased from 4.8 to 3.3.

**Table 2:** Variation in emotional state and difficulties.

Variables	Mean	Median	Standard deviation	Minimum	Maximum
Emotional State T1	6,89	8	2,814	1	10
Emotional State T2	6,75	7	2,754	1	10
Emotional State T3	7,16	8	2,65	0	10
Difficulties T1	4,8	4,5	2,898	1	10
Difficulties T2	4,45	5	2,619	1	10
Difficulties T3	3,3	4	2,646	0	9
EmoTICare coping support	7	8	2,901	0	10
EmoTICare help to emotional state	6,7	7,5	3,054	0	10
Specific Aid. 1st Area	6,91	7	2,752	0	10
Specific Aid. 2nd Area	6,64	6,5	3,012	0	10
Specific Aid. 3rd Area	6,93	8	3,038	0	10
Specific Aid. 4th Area	6,75	7,5	2,989	0	10
Specific Aid. 5th Area	6,95	8	3,004	0	10
Specific Aid. 6th Area	6,98	8	2,913	0	10

Additionally, the adolescents with T1DM rated emoTICare with an average of 7 and 6.7, respectively, on a scale ranging from 0 to 10, indicating the degree to which it has helped them cope with daily life challenges and improve their emotional state.

A thorough examination of the data reveals that adolescents perceive the sixth and fifth areas as the most beneficial, with the sixth area receiving the highest average score of approximately 7 out of 10. Notably, the eighth area also emerges as a frequent choice, with an average score of 8, suggesting a consistent preference among adolescents. In the subsequent section, the remaining points of results will be described and analyzed, with a focus on the profile of 73 participants who were assessed prior to the commencement of therapeutic work with emoTICare. This analysis will facilitate the identification of the challenges faced by this demographic, thereby substantiating the necessity for the implementation of programs such as emoTICare, in addition to the benefits previously outlined in this section of the results.

### 3.2 Sociodemographic variables

As illustrated in **Table 3**, the initial sample comprised 73 adolescents diagnosed with T1DM, of whom 64.4% were female. A significant proportion of the sample had a history of psychological consultation, with 58.9% reporting at least one such visit. Furthermore, 86.3% had been hospitalized at least once, highlighting the substantial clinical burden associated with the disease. With respect to their physical comorbidities, asthma, allergies, and epilepsy accounted for 5.5% each, while 79.5% of the subjects did not suffer from any other chronic disease in addition to T1DM. With regard to the presence of psychological comorbidities, anxiety (9.6%) and ADHD (8.2%) were the most prevalent, although the majority of the sample (80.8%) did not exhibit any psychological disorder. With regard to the most prevalent treatment for T1DM, our observations indicated that injectable insulin was the predominant approach (91.8%), followed by dietary management (39.8%). The physical consequences derived from this disease that were most frequently reported by the participants are visual difficulties (38.4%) and pain in the extremities (20.5%).

**Table 3:** Descriptive data on clinical and sociodemographic variables.

Clinical and Sociodemographic Variables		n	%
Gender	Male	26	35,6
	Female	47	64,4
Visited a psychologist at any point in time	Yes	43	58,9
	No	30	41,1
Hospitalized	Yes	63	86,3%
	No	10	13,7%
Physical Comorbidities	Asthma	4	5,5
	Allergy	4	5,5
	Epilepsy	4	5,5
	Others	8	11
	No	58	79,5
Type of treatment	Insulin Injections	67	91,8
	Insulin pump	7	9,6
	Feeding control	29	39,7
	Physical exercise	28	38,4
	Other	2	2,7
Psychological Comorbidities	Anxiety	7	9,6
	Depression	4	5,5
	TCA	2	2,7
	ADHD	6	8,2
	Others	1	1,4
	No	59	80,8
Physical Consequence of Diabetes	Vision difficulties	28	38,4
	Pain in hands or feet	15	20,5
	Skin problems	5	6,8
	Urination problems	1	1,4
	Problems with digestion	3	4,1
	Others	8	11
	No	36	29,7

As we can see in **Table 4**, the mean age of the sample was 14.49 years (SD = 1.69), with a minimum of 12 years and a maximum of 17 years. The adolescents reported a mean time since diagnosis of the disease of 61.68 months (SD = 44.83), with a wide range between 6 and 180 months. The mean number of hospitalizations due to the disease was 2.18 (SD = 2.04), with a substantial range between the minimum and maximum, 1 and 15, respectively. The frequency of visits to the endocrinologist due to T1DM averaged 3.16 months (SD = 1.76), with a minimum of two weeks between visits and a maximum of 12 months.

**Table 4:** Quantitative clinical descriptive data.

Variables	Mean	Standard deviation	Minimum	Maximum
Age	14,49	1,692	12	17
Time since diagnosis	61,68	44,83	6,00	180
Number of hospitalizations	2,18	2,04	1	10
Degree of difficulty of the disease	5,10	2,77	1	10
Emotional State Rating (0-10)	6,79	2,76	1	10
Frequency of visits to specialists (months)	3,16	1,76	,50	12

Finally, on a scale ranging from 0 to 10, the adolescents reported a mean of 5.10 (SD = 2.77) for the degree of difficulty posed by their disease and a mean of 6.79 (SD = 2.76) for their overall emotional state.

### 3.3 Profile of the variables of the 1st area: psychoeducation

In **Table 5**, we can observe the variables strongly related to physical well-being and the perception of threat from their illness. Regarding the perceived threat of the disease, the participants presented a mean of 30.9 (SD = 9.69) on the BIPQ scale. A comparison of these results with those from other studies employing this instrument in a similar population (patients aged 9–16 years with T1DM) reveals that the former are considerably higher.

**Table 5:** Profile of variables in the Psychoeducation area.

Questionnaire	Variable	*M*	Median	SD	P25	P50	P75	Minimum-Maximum	Questionnaire Range
BIPQ	Threat of illness	30,90	30,00	9,69	24	30	39	5-50	4-50
Kidscreen-27	Physical Well-Being	14,33	14,00	4,38	11	14	18	6-23	5-25

^M, Mean; SD, Standard Deviation; P25, 25th Percentile; P50, 50th Percentile; P75, 75th Percentile.^

The mean observed in this sample exceeds the 80th percentile (29.40) of the score obtained in studies with similar patients, suggesting that adolescents with T1DM in this sample have a high perception of disease. The percentiles further elucidate that a mere 25% of the sample has a score that is equal to or lower than 24, a point that can be identified as the “average” scores of the scale.

In the context of physical well-being, the mean direct scores of the sample were 14.33 (SD = 4.38). Given the scale of the test, it can be concluded that these scores are intermediate/low. A consultation of the scale of this instrument reveals that the mean is approximately the 32nd percentile, thereby confirming that the scores obtained by the adolescents in the physical well-being variable are deficient to a certain extent.

### 3.4 Profile of 2^nd^ and 3^rd^ area variables: emotional awareness and emotional regulation

**Table 6** presents the profile of the relevant variables related to the contents of this area. With respect to psychological well-being, the mean of the direct scores obtained is 23.63 (SD = 6.64). When evaluated on the standardized scale, the score obtained falls close to the mean.

**Table 6:** Profile of variables in the area of Emotional Awareness and Regulation.

Questionnaire	Variable	*M*	Median	SD	P25	P50	P75	Minimum-maximum	Questionnaire range
Kidscreen-27	Psychological Well-Being	23,63	25,00	6,64	20	25	29	8-35	5-35
EAQ	Distinguish Emotions	13,42	14,00	3,27	11	14	16	7-21	7-21
	Verbal exchange of emotions	5,51	5,00	1,87	4	5	7	3-9	3-9
	No concealment of emotions	9,25	9,00	2,23	8	9	11	5-15	5-15
	Body awareness	10,63	11,00	1,92	10	11	12	5-14	5-15
	Emotion analysis	11,88	12,00	2,11	11	12	14	6-15	5-15
	Attention to the emotions of others	11,64	12,00	1,12	11	12	12	9-15	5-15
	Emotional Awareness Total	62,33	62,00	6,45	57	62	67	46-78	30-90
SDQ	Emotional symptomatology	4,40	4,00	2,99	2	4	7	0-10	0-10
	Behavioral problems	3,27	3,00	2,08	2	3	5	0-8	0-10
	Hyperactivity	4,58	5,00	2,04	3	5	6	0-10	0-10
	Psychopathology and general adjustment	15,37	16,00	6,29	11	16	20	2-32	0-40

^M, Mean; SD, Standard deviation; P25, 25th percentile; P50, 50th percentile; P75, 75th percentile.^

With respect to the emotional awareness of the sample, it was observed that the sum of the subdimensions had a mean of 62.33 (SD = 6.45), which indicated that adolescents with T1DM exhibited an intermediate emotional awareness. Two of the most salient subdimensions were “attention to others’ emotions” and “analysis of emotions,” with 50% of the sample scoring above 12, approaching the upper limit of the dimension. Conversely, the sample scored low on “not hiding emotions,” with 50% of the sample scoring below 9.

A thorough examination of the subdimensions of the SDQ questionnaire reveals that the scale on which they achieve the highest scores is “hyperactivity,” with a mean of 4.57 (SD = 2.04). Conversely, “behavioral problems” emerges as the subdimension where they attain the lowest scores, with a mean of 3.27 (SD = 2.08). An examination of **Table 7**, which delineates the number of participants falling within each range specified by the instrument, reveals that the majority of participants are situated within the “normal” range across the subdimensions. However, when the overall scale score is considered, it becomes evident that 50.7% of the sample falls within the “borderline” and “clinically significant” categories with respect to their psychopathology and overall emotional adjustment. Concurrently, 41.1% of the participants exhibited “borderline” or “clinically significant” scores in the “behavioral problems” dimension, 37% in the “emotional symptomatology” dimension, and 30.2% in the “hyperactivity” subdimension.

**Table 7:** Subdimensions of “emotional psychopathology and behavioral problems” according to the scale (SDQ).

Variables	*M*	DT	Normal (%)	Borderline (%)	Clinically significant (%)
Emotional symptomatology (SDQ)	4,39	2,99	46 (63,3)	8 (11)	19 (26)
Behavioral problems (SDQ)	3,27	2,08	43 (58,9)	10 (13,7)	20 (27,4)
Hyperactivity (SDQ)	4,57	2,04	51 (69,9)	11 (15,1)	11 (15,1)
Psychopathology and general emotional adjustment (SDQ)	15,37	6,29	36 (49,3)	18 (24,7)	19 (26)

### 3.5 Profile of the variables of the 4^th^ area: coping and problem solving

This domain encompasses variables associated with decision-making and problem-solving capacity, as well as the ability to overcome the challenges that adolescents with T1DM may encounter. **Table 8** presents the results obtained for both the problem-solving variable, measured with the SPSI, and the resilience variable, measured with the BIPQ questionnaire, of adolescents with T1DM.

**Table 8:** Profile of variables in the problem solving area.

Questionnaire	Variable	*M*	Median	SD	P25	P50	P75	Minimum-maximum	Questionnaire range
SPSI	Rational towards problems	10,06	9	4,56	8	9	13	0-20	0-20
	Problem-avoidant	8,42	8	4,14	5	8	12	2-20	0-20
	Impulsive towards problems	7,98	7	4,26	5	7	11	0-18	0-20
	Positive Problem Orientation	10,06	9	4,76	7	9	14	0-20	0-20
	Negative orientation towards the problem	10,32	11	5,09	6	11	15	0-20	0-20
	Adaptive Resolution Factor	20,13	20	8,09	13	20	26	6-40	0-40
	Resolution Factor Maladaptive	26,73	26	11,44	17	26	35	4-54	0-60
CD-RISC	Resilience	22,20	23	8,21	17	23	28	0-40	0-40

M, Mean; SD, Standard Deviation; P25, 25th percentile; P50, 50th percentile; P75, 75th percentile.

The subdimensions of the SPSI scale indicate that adolescents have mean scores in both the “positive towards problems” (M = 10.06; SD = 4.76) and “negative towards problems” (M = 10.32; SD = 5.09) coping types, as well as the “rational towards problems” coping type (M = 10.06; SD = 4.56). Conversely, adolescents exhibited somewhat lower scores than the mean of the questionnaire in the types of coping characterized by avoidance (M = 8.42; SD = 4.14) and impulsivity (M = 7.98; SD = 4.26) when confronted with the occurrence of a problem.

This finding suggests that, while adolescents may engage in meticulous examination of the pros and cons prior to making significant decisions or attempting to resolve problems, a discrepancy exists in their approach to decision-making, as evidenced by the comparable utilization of both negative and positive orientations.

Regarding resilience, the adolescents obtained a mean of 22.20 (SD = 8.21), which could be identified as an intermediate score given the range of possible scores of the instrument, although it is also observed that 25% of the sample has scores below 18.50, a fact that reveals that certain participants have difficulties in adapting to adverse situations in an adaptive manner.

### 3.6 Profile of the variables of the 5^th^ area: identity

This section encompasses the variables that pertain to the self-concept, both physical and psychological, of the subjects. That is to say, the variables that indicate the perceptions of adolescents with T1DM regarding themselves and their own abilities. **Table 9** below presents the profile of the adolescents in the self-concept variable.

**Table 9:** Self-concept variable profile (CAG).

Questionnaire	Variable	*M*	Median	SD	P25	P50	P75	Below the standardized 50th percentile (%)	Above the standardized 50th percentile (%)	Minimum-maximum	Questionnaire range
CAG	Physical Self-Concept	28,63	29	4,87	25	29	32	29 (39,7)	44 (60,3)	15-38	8-40
	Social Self-Concept	26,88	28	6,42	24	28	32	46 (63)	27 (37)	12-38	8-40
	Family Self-concept	28,90	30	5,50	26	30	34	24 (32,9)	49(67,1)	16-38	8-40
	Intellectual Self-Concept	26,38	26	5,79	21	26	32	34(46,6)	39 (53,4)	16-38	8-40
	Personal Self-Concept	27,42	29	4,52	25	29	31	55 (75,3)	18 (24,7)	17-38	8-40
	Control capacity	25,10	24	5,17	21	24	29	47 (64,4)	26 (35,6)	16-37	8-40
	General Self-Concept	163,32	163	23,55	147	163	181	–	–	107-204	48-240

The Garley self-concept questionnaire yields a set of scaled scores that facilitate the comparison of our sample’s scores with a reference score (see **Table 9**). As demonstrated in the results, the subdimension in which the adolescents demonstrate the highest mean scores is family self-concept (M = 28.90; SD = 5.50). A total of 67.1% of the sample exceeds the 50th percentile of the standardized scale in this domain. The subdimension in which they present more problems is personal self-concept (M = 27.42, SD = 4.32), where 75.3% of the sample is below the 50th percentile of the scale. Other dimensions that merit attention for their substandard scores include control capacity (M = 25.10, SD = 5.17) and social self-concept (M = 26.88, SD = 6.42), with 64.4% and 63% of the sample, respectively, falling below the 50th percentile of the standardized scale.

### 3.7 Profile of variables in the 6^th^ area: social skills

Finally, this section groups together the variables referring to the social skills and prosocial behavior of adolescents with T1DM, as well as their close relational framework, in which we can include their family relationship and school performance (**Table 10**).

**Table 10:** Profile of the variables in the Social Skills area.

Questionnaire	Variable	*M*	Median	SD	P25	P50	P75	Minimum-maximum	Questionnaire range
CHASO	Interacting with strangers	9,49	9	3,70	7	9	12	4-20	5-20
	Express positive feelings	14,27	14	4,78	11	14	19	4-20	5-20
	Facing Criticism	13,38	14	4,30	11	14	17	4-20	5-20
	Interact with significant others	9,73	9	4,87	6	9	13	4-20	5-20
	Calmness in the face of criticism	12,44	12	3,96	10	12	15	4-20	5-20
	Public Speaking	11,05	11	3,64	8	11	14	4-20	5-20
	Facing ridicule	9,60	10	3,29	8	10	12	4-18	5-20
	Defense of rights	12,51	12	3,79	10	12	15	5-20	5-20
	Ability to Apologize	14,84	15	4,26	12	15	18	4-20	5-20
	Rejection of requests	14,84	15	4,26	12	15	18	4-20	5-20
	Total Social Skills	122,15	118	26,85	107	118	143	57-192	50-200
Kidscreen-27	School Welfare	13,68	14	3,26	12	14	16	5-20	5-20
	Family relationship and autonomy	24,15	25	5,82	20	25	28	12-35	7-35
	Social Support	15,63	16	3,47	13	16	19	4-20	5-20
SDQ	Prosocial Behavior	7,66	8	1,85	6	8	9	3-10	0-10
	Problems in relationships with peers	3,12	3	1,91	2	3	4	0-10	0-10

M, Mean; SD, Standard Deviation; P25, 25th percentile; P50, 50th percentile; P75, 75th percentile.

In consideration of the dimensionality of the CHASO questionnaire, it was determined that adolescents exhibit the lowest scores in the interaction domain, encompassing interactions with both acquaintances and individuals of a romantic or sexual interest. This observation is further substantiated by the observation that both categories exhibit comparable means, with a mean of 9.73 (SD = 4.87) in the context of peers and a mean of 9.49 (SD = 3.70) in the context of acquaintances of a romantic or sexual interest. The subjects demonstrated particularly strong aptitude in apologizing and declining requests, with a mean score of 14.84 and a standard deviation of 4.78. This was followed by the expression of positive emotions, which exhibited a mean score of 14.27 and a standard deviation of 4.78.

The mean (M) and standard deviation (SD) of the subdimensions of the social skills scale, as well as their overall score (M = 122.15; SD = 26.85), could be considered as somewhat below average when compared with other applications of the questionnaire, especially with regard to their ability to interact with people they find attractive, in which 25% of the sample presents low scores (scores below 6). The scores on the dimensions of the Kidscreen-27 questionnaire were found to be lower than the mean of the official scales of the instrument. Specifically, the mean score pertaining to the relationship with parents and relatives would be at the 30th percentile on the standardized scales of the instrument. This suggests that these adolescents may encounter difficulties in establishing boundaries and may not perceive their parents as providing sufficient autonomy (M = 24.15; SD = 5.82).

**Table 11** presents the percentage and number of subjects who fall into each of the ranges established by the SDQ instrument for the variables of prosocial behavior and problems in relationships with peers. As demonstrated in the data, a significant proportion of the sample falls within the category of borderline or clinically significant scores for both variables (11% and 35.7%, respectively). However, the majority of these scores are within the “normal” range in these subdimensions.

**Table 11:** Subdimensions of the variables “prosocial behavior” and “relational problems with peers” according to the scale (SDQ).

Variables	*M*	DT	Normal (%)	Borderline (%)	Clinically significant (%)
Prosocial Behavior	1,16	0,5	65 (89)	4 (5,5)	4 (5,5)
Relationship problems with peers	1,46	,68	47 (64,4)	18 (24,7)	8 (11)

M, Mean; SD, Typical Deviation.

### 3.8 Profile of the health-related quality of life variable

Although we have distributed its different subdimensions throughout the areas, we consider that the health-related quality of life variable deserves to be considered globally, given that it is one of the main factors in assessing adaptation to the disease. In **Table 12** we can observe the mean T scores (the previous ones were the direct scores of the instrument, without transformation) of each of the subscales and their comparison with the 50th percentile of the European reference scale, which will allow us to make a more precise interpretation of these.

**Table 12:** Health-related quality of life, comparison with standardized 50th percentile.

Variable	*M*	SD	Lower than 50th percentile N (%)	Above the 50th percentile N (%)
Physical Well-Being	41,96	10,66	56 (76,7)	17 (23,3)
Psychological Well-Being	41,15	10,98	58 (79,5)	15 (20,5)
Family Relationship	44,22	9,41	62 (84,9)	11 (15,1)
Social Support	48,02	10,93	40 (54,8)	33 (45,2)
School welfare	45,96	9,45	52 (71,2)	21 (28.8)

M, Mean; SD, Standard Deviation.

A subsequent analysis of the scores obtained in the different subdimensions of quality of life evaluated with the Kidscreen questionnaire revealed that the majority of the sample is below the 50th percentile in all subdimensions. This finding was then compared with the standardized scales corresponding to their age group and sex. Specifically, the dimensions of “Psychological Well-being” and “Family Relationship” demonstrate the most significant impact, with 79.5% and 84.8% of the sample falling below the 50th percentile, respectively.

### 3.9 Main correlations between the study variables

As illustrated in **Table 13**, the primary correlations of the study were determined using Spearman’s Rho statistic, given that certain variables did not satisfy the assumption of normality.

**Table 13:** Correlation matrix of the main variables of the study.

Variables	1	2	3	4	5	6	7	8	9	10	11	12	13	14	15
1.Psychopathology and general emotional adjustment	1,000														
2. Total Social Skills	0,206	1,000													
3.Total Self-Concept	-,453**	,316**	1,000												
Emotional Awareness	-,306**	,233*	,477**	1,000											
5.Resilience	-,358**	,281*	,522**	0,215	1,000										
Perceived threat of illness	,619**	0,113	-,422**	-,341**	-,461**	1,000									
Physical Well-Being	-,414**	,287*	,631**	,458**	,415**	-,402**	1,000								
8. Psychological Well-Being	-,611**	0,013	,457**	,425**	,343**	-,497**	,604**	1,000							
9. Family Relationship	-,423**	,259*	,596**	,489**	,381**	-,360**	,594**	,635**	1,000						
10. Social Support	-0,220	,368**	,426**	,346**	,412**	-,296*	,368**	,464**	,485**	1,000					
11. School well-being	-,253*	,531**	,710**	,411**	,489**	-,290*	,699**	,473**	,602**	,447**	1,000				
12. Health-Related Quality of Life	-,469**	,369**	,700**	,519**	,500**	-,462**	,829**	,772**	,804**	,694**	,799**	1,000			
13. Age	-0,103	-0,092	0,154	0,135	-0,081	0,021	-0,121	-0,118	0,037	-0,098	-0,110	-0,103	1,000		
14. Adaptive problem solving style	-0,062	,462**	,436**	0,122	,422**	-0,045	,271*	0,102	,245*	0,182	,344**	,296*	-0,028	1,000	
15. Disadaptive problem-solving style	,551**	,236*	-,333**	-,406**	-0,212	,366**	-,398**	-,417**	-,382**	-0,118	-,274*	-,401**	-0,111	0,198	1,000

^*= p<0.05,** p<0.01.^

The results of the correlation matrix in reveal a negative correlation between the presence of psychopathology and negative emotional adjustment (SDQ) and total self-concept (ρ = -.453, p <.01), resilience (ρ = -.358, p <.01), and psychological well-being (ρ = -.611, p <.01). The correlation is also negative with the variable health-related quality of life, which is the sum of all subdimensions of the Kidscreen-27 questionnaire (ρ = -.469, p <.01). This finding suggests that adolescents grappling with significant emotional challenges may also exhibit diminished self-confidence, weaker coping mechanisms, and a diminished perception of overall quality of life when confronted with life’s challenges.

Conversely, heightened perceived illness threat (BIP-Q) exhibited a strong correlation with the presence of psychopathology (ρ= .619, p <.01) and a diminished capacity for resilience (ρ= -.461, p <.01). These findings imply that the negative perception of T1DM exerts a direct influence on emotional well-being and the individual’s capacity to effectively manage its challenges. Individuals with a higher perception of illness also tend to adopt maladaptive coping styles in response to problems (ρ = .366, p <.01).

The findings revealed a significant positive correlation between total self-concept score and psychological well-being (rho = .631, p <.01), health-related quality of life (rho = .700, p <.01), and adaptive coping style toward problems (rho = .436, p <.01). Conversely, lower self-concept demonstrated a negative correlation with maladaptive coping style (rho = -.333, p <.01) and elevated perceived threat to illness (rho = -.422, p <.01).

No significant correlations were observed between participants’ age and their perception of illness threat, resilience, or social skills. However, a low, but not significant correlation was identified between age and health-related quality of life (ρ = -.103).

### 3.10 Comparison according to gender

A comparative analysis was conducted of the scores on the primary psychological variables of the study, categorized according to the gender of the adolescents. The results of this analysis are presented in **Table 14**. Due to the fact that certain variables did not satisfy the assumption of normality necessary for the Student’s t-test to be employed, a mixed analysis was deemed the optimal approach. Consequently, the variables that did not meet this assumption were subjected to analysis using the Mann-Whitney U test. The identification of these non-normal variables is facilitated by the data of average ranges, denoted by the symbol/*/Rp///.*

**Table 14:** T-test comparison according to gender.

Questionnaire	Variable	Gender		t/Z	p	d/r
		Male *//Ar///M (DT) (n=26)*	Female *//Ar//M (DT) (n=47)*			
SDQ	Emotional Symptomatology	*//33,19//*3,92 (3,92)	*//39,11//*4,65 (2,87)	1,146	,252	,134
	General psychopathology	15,03 (7,21)	15,55 (5,78)	-,188	,851	-,081
CHASO	Interacting with people they are attracted to	*//44,23//*11,54 (5,29)	*//33//8*,72 (4,36)	2,177	,030*	,255
	Public speaking	12,65 (3,61)	10,17 (3,38)	2,932	,009**	,717**
	General social skills	129,19 (28,31)	118,26 (25,47)	1,688	,096	,413
Kidscreen-27	Physical Well-Being	47,44 (10,71)	38,93 (9,44)	3,516	<,01**	,859**
	Psychological well-being	44,73 (12,72)	39,18 (9,47)	2,115	,038*	,517*
	Family Support	*//42,63//*47,04 (10,45)	*//33,88//*42,65 (8,49)	-1,691	,091	,198
	Social Support	*//39,94//*49,21 (12,70)	*//35,37//*47,37 (9,91)	-,887	,372	-,104
	School well-being	*//44,85//*49,21 (9,54)	*//32,66//*44,16 (9,00)	-2,364	,018*	-,277
	Total HRQoL	244,16 (38,43)	217,05 (35,57)	2,616	,011*	,738**
CAG	Total self-esteem	167,38 (22,31)	161,06 (24,15)	1,100	,275	,565*
EAQ	Body Consciousness	*//31,90//*10,15 (2,07)	*//39,82//*10,89 (1,80)	1,594	,121	,187
	Emotion Analysis	*//44//*12,50 (2,16)	*//33,13//*11,53 (2,03)	-2,121	,034*	-,248
SPSI	Positive Problem Orientation	*//44//*11,62 (5,27)	*//33,13//*9,21 (4,29)	-2,103	,035*	-,246
	Negative orientation towards the problem	*//33,27//*9,62 (5,78)	*//39,06//*10,72 (4,69)	1,121	,262	,131
	Rational attitude towards problems	*//42,52//*11,46 (4,73)	*//33,95//*9,30 (4,33)	-1,663	,096	-,195
	Adaptive coping style	*//44,12//*23,08 (8,25)	*//33,06//*18,51 (7,61)	-2,134	,033*	-,250

/*/Ar//*, average ranges; M, mean; SD, standard deviation; Z/, value of Mann Whitney U test statistic or t-test; p, level of significance *p ≤0.1 **p ≤0.05 and ***p ≤0.01; r/d=effect size (in r 0.1-0.3=weak effect, 0.3-0.5*=medium effect, >0.5**=large effect) (in Cohen’s d = small TE ≈ 0.20; moderate TE ≈ 0.50; large TE≈ 0.80.

A comparison of male and female subjects reveals that males exhibit higher scores in the following subdimensions related to quality of life: physical well-being (p <.01); school well-being (p <.05); and psychological well-being (p <.05). However, no significant differences were observed in the dimensions of family relationships and social support. The high effect size (d=.859) for the physical well-being variable and moderate effect size for the psychological well-being variables (d=.517) should be highlighted. With respect to the social skills subdimensions, significant differences were observed in the subdimensions of “public speaking” (p <.01) and “interacting with people they are attracted to” (p <.05), with high (d = .717) and moderate (r = .255) effect sizes, respectively.

With regard to emotional awareness, substantial disparities are evident in the subdimension of emotion analysis, wherein male subjects demonstrate significantly higher scores compared to female subjects (p <.05), exhibiting an effect size approaching moderate (d = -.248). A significant disparity emerges in the context of their approach to problem-solving, with male subjects exhibiting a pronounced inclination toward a positive orientation in problem-solving (p <.05). Conversely, boys exhibited a higher adaptive attitude toward problem-solving than girls (p <.05). The effect sizes for these differences are relatively moderate in the case of adaptive style (r=-.250) and positive problem orientation (r=-.246).

After thorough examination, it was determined that there were no substantial disparities observed in the total self-esteem score, the emotional symptomatology exhibited by adolescents, or the potential presence of psychopathologies.

## 4 Discussion

The main objective of this study was to evaluate the effects of an intervention designed for adolescents with T1DM, implemented through the use of a serious game called emoTICare.

This analysis was conducted to evaluate the strength of the evidence supporting our hypotheses. The emoTICare program has been shown to positively impact the clinical and emotional health indicators of adolescents with T1DM and has the potential to further improve these indicators.

A substantial decline in the perceived threat of illness was observed following the utilization of the serious game, the variable that exhibited the most significant discrepancy within the sample. In terms of resilience, a perceptible increase was detected at T2-T3, accompanied by a slight reduction in emotional symptomatology. This finding aligns with the conclusions of previous reviews, which have emphasized the effectiveness of serious games in enhancing resilience among adolescents with T1DM. Recent pilot studies have validated the viability of mobile game-based interventions in augmenting coping resources and resilience (65, 66).

Measures of social skills showed positive trends in “interacting with strangers,” “public speaking,” and “coping with ridicule” following the game experience. Given that social interaction is one of the main stressors in this population (67), intensify the components of assertive communication and conflict resolution, reinforcing the social support mechanisms that have been shown to improve the quality of life in adolescents with T1DM (68).

The self-concept analysis further demonstrated a favorable trend across all subdimensions of the scale subsequent to the implementation of emoTICare. Self-concept is a significant predictor of adjustment in adolescents with chronic diseases, including T1DM. A body of research indicates a correlation between a positive self-concept and enhanced glycemic control, as well as elevated levels of self-care behaviors. This notion is further substantiated by the findings of a study conducted by Kenowitz et al. (69) found a significant correlation between diabetes-specific self-esteem and self-care as well as HbA1c levels.

A favorable trend has also been observed in emotional awareness, defined as the degree to which participants can identify and articulate their feelings after utilizing emoTICare. This factor is of particular significance because, in adolescents with T1DM, the capacity to recognize and manage one’s emotions has been linked with enhanced adherence to treatment and more stable glycemic control. This association is attributed to the facilitation of more deliberate coping with the emotional challenges associated with the disease (70). As we have seen, the most robust effects appeared on perceived illness threat and on emotion-related competencies—notably the verbal exchange of emotions—with resilience also showing meaningful improvement, whereas broader or more distal constructs (e.g., global HRQoL, self-concept, overall social skills) showed only positive but nonsignificant trends. This might mean that some of the skills that we are aiming to improve with the intervention manifest a meaningful change over shorter windows, whereas identity-laden or contextual outcomes (self-concept, HRQoL, family/school functioning) often require longer consolidation and/or changes in the adolescents’ environments to register statistically (our own limitation note highlights that the six-week intervals may be insufficient to capture deep changes in complex affective competencies and self-concept).

In the same way, we might not see sufficient change in these variables due to a varied measurement precision across subscales; for example, several EAQ-30 subdimensions in our sample showed modest reliability (e.g., very low α for bodily awareness), which would dampen sensitivity to change. Third, some SDQ domains improved only between T2 and T3—the active exposure window—suggesting that effects are time-locked to engagement and may need longer or booster dosing to generalize to broader functioning.

If we take a look at previous literature, feasibility triаls оf smartphоne-based sеrious games havе similarly reported proximal gains in resiliency and coping resources following short-term engagement, underscoring the potential оf gamified approaches to strengthen immediate psychosocial skills in this population (65).

Systematic reviews оf psychological and psychoeducational interventions further indicate that while such programs often produce meaningful changes in distress, coping, and emotional regulation, their effects on global quality оf life and objective clinical outcomes (e.g., HbА1c) are less соnsistent, particularly in studies with brief intervention windows or modest sample sizes (71).

This distinction bеtwееn proximаl аnd distal outсomes is аlso highlighted in thе litеraturе оn rеsiliеncе- аnd emоtiоn-fоcused trаining. Interventiоns expliсitly tаrgeting rеsiliеncе havе been shown to imрrove strеss managеmеnt, self-efficacy, аnd psyсhosoсial adjustment, yet rеquirе lоngеr follow-up оr booster sessiоns to cоnsolidаte broadеr impacts оn well-being аnd quality оf life (72).

By cоntrast, self-cоncept, fаmily relatiоnships, аnd glоbal НRQoL arе mоre comрlex, idеntity- аnd envirоnment-driven cоnstructs, whiсh may rеquirе sustаined рractice аnd broadеr systemic suppоrt (fаmily, sсhool, healthcarе sеttings) to demоnstrate meаsurаble imрrovement (73). Taken together, the significant improvements in resilience and emotional awareness are theoretically coherent and consistent with prior literature, whereas nonsignificant changes in more distal outcomes are plausibly explained by the shorter follow-up, slightly underpowered final sample, reliability constraints in some measures, and the need for longer or ecosystem-level supports to translate proximal skills into global quality-of-life gains. Following the intervention results, we also explored the different psychosocioemotional characteristics of a sample of adolescents with T1DM in order to justify the importance of psychological interventions in this population. The DAMIP model was developed by synthesizing several models related to chronic disease (33), that try to elucidate the factors that influence adolescents’ ability to adapt to chronic diseases.

We have subsequently investigated the psychosocioemotional profile of the variables associated with the domains delineated in the DAMIP model, with the objective of assessing the extent of participant involvement in each domain. The objective of this study is to elucidate the psychosocial and emotional challenges experienced by adolescents with type 1 diabetes mellitus (T1DM) and to provide a comprehensive profile of the psychological and social functioning of this vulnerable population.

At the baseline assessment of the cross-sectional cohort (N = 73), adolescents with T1DM exhibited elevated disease threat perception, which exceeded the 80th percentile of comparable samples (44). Concurrently, the indices of physical well-being were situated around the 32nd percentile on the European scale. Notably, more than 75% of the young people demonstrated scores below the 50th percentile in the domains of psychological well-being and family relationships, signifying a pronounced deficit in these areas. The presence of emotional and behavioral symptomatology was notable: 39.5% of the participants obtained scores on the SDQ questionnaire that were classified as “borderline” or “abnormal.” These data reveal critical areas of vulnerability, especially the perception of threat of illness and emotional well-being. Concurrently, studies have identified a correlation between living with T1DM and elevated stress levels, which, in many cases, results in reduced adherence to treatment. This can lead to a deterioration in glycemic control among affected adolescents (74). In turn, it is common to find high levels of general and specific discomfort related to diabetes (75) in addition to co-morbid psychological problems - such as depression, anxiety (76)– or behavioral problems (8, 23). These difficulties can ultimately have a negative impact on the quality of life and glycemic control of those suffering from it (77).

The results also reflect the existence of a somewhat improvable repertoire of social skills, with particular difficulties in areas such as “Interacting with strangers” and “Interacting with significant others.” These results indicate that the subjects do not feel capable of interacting correctly with strangers and with those to whom they feel some kind of attraction. The cultivation of these competencies is of paramount importance during this developmental stage, as adolescence is characterized by an escalating significance of the social sphere. This is primarily driven by the heightened value attributed to peer influence and social approval (78, 79). Many adolescents encounter challenges in establishing and maintaining effective interpersonal relationships (80), which can result in a deterioration of their socioemotional state (81). In this sense, some studies, like the one made by Chao et al. (67) indicate that this is a critical factor for adolescents with T1DM, as it has been identified as the second most significant stressor in this demographic.

With regard to resilience, 25% of the sample exhibited values below 17, which can be defined as low. This is of great importance because resilience has been identified as a key protective factor for mental health in the context of chronic disease. Indeed, research has demonstrated that resilience mediates the effects of disease-related stress on psychological outcomes (82). Therefore, it is imperative to consider this indicator when implementing interventions designed to affect the mental well-being of adolescents with T1DM.

An examination of the scores on the self-concept variable reveals results that may be of concern. For instance, 75.3% of the sample falls below the 50th percentile on the personal self-concept subscale, and 63% of the sample falls below the 50th percentile on the social self-concept subscale. Self-concept is a construct closely related to self-identity, the construction of which is accentuated during the period of adolescence (83). Thus, an adjusted self-concept can promote proper adjustment to the disease and improve quality of life in adolescents with T1DM (84, 85). A salient feature of the present study is the pronounced gender disparities observed in the sample. The data reveal that girls have lower self-concept scores, although the differences are not significant. These data align with other studies that have identified comparable gender disparities, as evidenced by the research conducted by Kenowitz et al. (69), which found lower self-esteem scores in girls with T1DM. The problem-solving skills of the sample of adolescents with T1DM are somewhat deficient, although it is observed that the scores of the sample with respect to the type of approach to problems lean toward the rational style, rather than other less adaptive styles, such as impulsive or avoidant. Despite this, adolescents are similarly inclined to view problems negatively as well as positively, as evidenced by similar means on the variables of negative orientation and positive orientation to the occurrence of problems, reflecting a clear ambivalence. In this variable, again, gender differences are again observed, as boys tend to deal rationally and with a more positive orientation to the occurrence of problems than girls. This is in line with other recent research in which adolescent girls had lower perceived self-efficacy in decision making than boys (86).

The mean scores for the perceived threat of the disease, in this case, T1DM, can be classified as moderate-high, indicating that the adolescents in the sample are profoundly concerned about the problems derived from their disease and may possess a series of maladaptive beliefs related to it. It is imperative to acknowledge the heightened perceived threat of disease among this demographic, as evidenced by numerous studies that demonstrate a correlation between disease perception and diminished quality of life, accentuated anxious-depressive symptomatology, and suboptimal treatment adherence (87, 88).

The results of the study, as measured by the Kidscreen-27 questionnaire, indicate that the majority of the sample falls below the mean in all dimensions analyzed by this instrument. This finding suggests that the majority of the sample exhibits suboptimal quality of life. Specifically, the highest percentages of subjects below the mean are in the dimensions of “psychological well-being” and “family relationship.” These findings appear to align with the extant scientific literature, which suggests that adolescents with T1DM are more susceptible to psychological disorders, such as anxiety and depression (89, 90), and that the difficulty of the family relationship may deteriorate as a consequence of the presence of a chronic disease (21).

In view of the aforementioned points, the findings of our study demonstrate the tangible benefits of the emoTICare platform on both the physical and psychological well-being of the subjects.

However, as we have discussed earlier, the study is not without its limitations, which may have influenced the results obtained and thus the scope of the intervention. First, the small final sample size (44 аdolescents) and recruitment from a single setting limit statistical power and generalizability and also mаy diminish stаtisticаl powеr to detect smаll effects, аnd increаsed thе risk оf Tyрe II errоrs. However, a priоri powеr аnаlysis (G*Рower 3.1) indicаted а required minimum оf 46 pаrticipаnts fоr detecting medium effects in repeаted-meаsures ANOVA, so thе diffеrenсe between thе cаlculаted аnd аctuаl sаmple sizе wаs relаtively smаll. The short follow-up period also can restrict conclusions about long-term effects of the intervention. Outcomes were based largely on self-report, which may be influenced by recall or social desirability bias, and fidelity of intervention delivery was not systematically assessed. Finally, dropout is а сommon chаllenge in longitudinаl studies with аdolescent populаtions, pаrticulаrly when pаrticipаtion requires repeаted online аssessments аnd sustаined engаgement over severаl weeks, and, in our case, may have introduced dropout bias. Тhis level оf аttrition mаy hаve introduced biаs, аs individuаls who discontinued pаrticipаtion cоuld diffеr systemаticаlly from thоse who remаined, fоr exаmple in terms оf motivаtion, diseаse mаnаgement, or psychosociаl functioning. We keep in mind that this was conducted as a pilot study, with acknowledged limitations pertaining to its statistical power. This error calls for implementing retention strаtegies in future triаls to enhаnce both internаl vаlidity аnd representаtiveness оf thе tаrget populаtion. The six-week interval between evaluations may prove insufficient to verify the consolidation of profound changes in complex affective competencies or constructs such as self-concept or physical and psychological well-being that could be generated by the appearance of emoTIcare. Therefore, it would be advantageous to generate new follow-up measures beyond T3 that facilitate the formulation of more definitive conclusions.

## 5 Conclusion

The present study underscores the psychosocial vulnerabilities and profile of chronically ill adolescents, encompassing their emotional difficulties, social and coping skills, emotional awareness, resilience, and the overall quality of life they experience. The findings underscore the considerable promise of the serious game emoTICare in diminishing threat perception, fostering resilience, and enhancing trends in self-concept and social skills. The primary benefits of the innovative and comprehensive emoTICare intervention, which integrates therapeutic work with five significant indicators of health-related quality of life (i.e. physical well-being, cognitive coping, emotional well-being, social relationships and support, and identity), are that it is also supported by a theoretical model, the DAMIP (33). The emoTICare intervention and its theoretical framework can serve as a guide for subsequent interventions aimed at enhancing health-related quality of life (hrQoL) in adolescents. These interventions may seek to replicate or improve the study’s results, thereby circumventing the substantial methodological and theoretical heterogeneity characteristic of many such interventions. Furthermore, as a serious game, emoTICare is distinguished by its scalability, as it can be applied to large samples without the need for direct intervention by professionals. These results emphasize the importance of comprehensive, multidisciplinary interventions and tools such as “emoTICare” that aim to strengthen psychosocioemotional resources in adolescents with T1DM to facilitate better adaptation to this chronic disease.

## Data Availability

The raw data supporting the conclusions of this article will be made available by the authors, without undue reservation.
